# A hybrid lamination strategy for enhanced performance in linear oscillation actuators

**DOI:** 10.1371/journal.pone.0360739

**Published:** 2026-10-26

**Authors:** Hongbin Zhang, Zhan Shen, Zhike Xu

**Affiliations:** 1 Jiangsu Maritime Institute, Nanjing, Jiangsu, China; 2 School of Electrical Engineering, Southeast University, Nanjing, Jiangsu, China; University of Oxford, UNITED KINGDOM OF GREAT BRITAIN AND NORTHERN IRELAND

## Abstract

Tubular permanent magnet linear oscillation actuators (TPMLOAs) are widely used in refrigeration, yet traditional radial lamination of silicon steel faces challenges with low stacking coefficients and assembly difficulties. This study proposes a hybrid lamination strategy that embeds silicon steels into fan-shaped soft magnetic composite (SMC) slots to optimize the stator core, aiming to combine the high permeability of silicon steel with the structural flexibility of SMC for a stacking coefficient near unity. A geo-metric model was established to derive material proportions, and 3-D finite element analysis was employed to investigate the effects of silicon steel quantity on electro-magnetic thrust, iron losses, and efficiency. The results indicate that the hybrid lamination method effectively suppresses magnetic saturation during load operation. Compared to traditional radial lamination, the proposed design increases electromagnetic thrust by 26% at the maximum mover displacement (where magnetic saturation is most severe), while thrust performance is comparable at the negative mover position. This thrust enhancement is particularly valuable for compressor applications, where high thrust at maximum displacement is critical. Although the inclusion of SMC increases eddy current losses at high frequencies, the method significantly simplifies manufacturing and reduces assembly errors. It is concluded that the hybrid lamination strategy provides a feasible and high-performance solution for the development of advanced linear oscillation actuators in compressor applications.

## 1. Introduction

Refrigeration compressors rely on reciprocating linear motion to compress refrigerant gas, which is conventionally driven by rotary motors via crankshaft mechanisms—introducing mechanical losses, wear, and noise. Linear oscillation actuators (LOAs) directly convert electromagnetic force into linear reciprocating motion, eliminating intermediate transmission parts. This enables compact, high-efficiency, and low-noise compressor designs, widely adopted in household and commercial refrigeration systems [1,2]. Due to the natural compatibility between the linear oscillating actuators and the piston structure of the compressor, the integrated design of the motor and com-pressor can be achieved, greatly simplifying the transmission process. Compared with traditional low-speed high-thrust linear machines [3,4], the tubular permanent magnet linear oscillator actuator (TPMLOA) performs particularly well in high-frequency linear reciprocating motion scenarios due to its high utilization of winding coils and permanent magnets (PM), simple mechanical structure, and com-pact size. Meanwhile, due to the closed-loop magnetic circuit of the TPMLOA being located on the axial section of the stator, it is necessary to use radial lamination of silicon steels to reduce magnetic reluctance. However, this stacking method is limited by the circular ring structure, resulting in a stator stacking coefficient of only 0.7, making it difficult to assemble and position the silicon steels. Therefore, developing an efficient lamination method suitable for TPMLOA has become a key issue that urgently needs to be overcome in this field.

For compressor operation, the maximum mover displacement (power stroke) is the most critical point: it requires peak thrust to overcome high refrigerant pressure, while severe magnetic saturation occurs due to superimposed armature and permanent magnet fields. Traditional silicon steel radial lamination suffers from low stacking coefficient, assembly difficulty, and aggravated saturation—limiting thrust output. Although the magnetic permeability of soft magnetic composite materials (SMC) is relatively low, it has significant advantages in achieving overall stator processing and improving the lamination coefficient [5–7]. In [5] and [6], a quasi-Halbach magnetized TPMLOA with stator made of SMC material is proposed. Its working characteristics are first predicted through analytical methods and comprehensively verified through finite element simulation and experimental testing. Meanwhile, the resonant frequency and efficiency of the TPMLOA is tested. Experimental results show that when the system reaches the resonant frequency of 44.5 Hz, the efficiency exceeds 93%. The eddy current loss of soft magnetic composite materials is relatively low at low frequencies [7]. However, due to the relatively high cost of soft magnetic composite materials, they are usually not given priority consideration when processing motors.

Given the cost limitations of SMC materials, in order to further improve the stator lamination coefficient, some scholars have shifted their research focus to optimizing traditional lamination methods and proposed a new type of LOA structure. In [8], a novel flux reversal linear oscillatory motor is proposed, with stator made of silicon steels stacked axially. The lamination method for the stator and mover cores is identical to that of conventional rotating motor cores, facilitating easy manufacturing and reducing costs. In [9], a stator-magnet moving-iron transversal-flux linear machine is developed to improve material utilization, reduce costs, and increase thrust density. The experimental results indicate that the proposed linear machine can drive cylinder with a relatively high discharge pressure [10].

In addition to improving the overall lamination method, some scholars have also proposed more refined assembly optimization schemes for the radial lamination characteristics of silicon steels. For example, by using a new method of separating the teeth and yoke of the outer stator from the laminations, the lamination coefficient can be in-creased from the initial 0.62 to 0.75 [11]. Based on the above research, a more complex lamination method is proposed in [12]. This hybrid lamination method stacks the stator teeth radially and the stator yoke axially according to the direction of magnetic circuit operation. In [13], a hybrid iron core is used to design a flux-switching TPM-LOA, and different amounts of silicon steels and SMC materials are given based on different frequencies. The hybrid stacking method is not only used in linear oscillating motors. In [14], a permanent magnet claw motor using a hybrid silicon steel and SMC is proposed. Research has shown that claw pole motors using hybrid laminated and soft magnetic composite materials have higher structural strength, larger output torque, and higher low-speed efficiency than traditional material claw pole motors.

At present, scholars have proposed various calculation methods for analyzing the electromagnetic characteristics of LOAs [15–18]. Regarding the lateral force problem of LOA, the response surface methodology is used to optimize the parameters of the PM, achieving a maximum reduction of 53.89% in lateral force while ensuring thrust [15]. Furthermore, the operating characteristics of LOA with mechanical loads are analyzed using 2-D FEA. And the efficient operating characteristics of electrical and natural resonance frequency matching are verified through experiments [16]. To ensure the accuracy of the analytical model, finite element analysis often requires close integra-tion with experimental validation, which aligns with the research methodology in [17,18] that verified the effectiveness of health monitoring algorithms through con-structed experimental platforms. A new design method has been proposed for a 3-kW single-phase linear permanent magnet generator (SPLPMG). The 2D FEA introduces the core laminations coefficient in the calculation to optimize the design and improve the accuracy of the results. Combined with tuned capacitor compensation winding inductance, a 3046 W output power is achieved at a driving frequency of 27 Hz. The experimental results have verified the feasibility of the design scheme [19]. A method for calculating iron loss based on 2-D axisymmetric FEA is proposed to address the is-sue of high computational cost caused by the circumferential segmented structure. The stator magnetic flux density is corrected using equivalent coefficients, effectively compensating for the influence of segmented structure on magnetic field distribution, and significantly reducing design costs while ensuring calculation accuracy [20].

In this paper, a hybrid lamination method is proposed to optimize the TPMLOA. The work commences with an overview of the TPMLOA’s fundamental structure, followed by a magnetic circuit analysis that informs the hybrid stator assembly strategy and determines the proportional use of SMC and silicon steel materials. Subsequent 3-D FEA simulations investigate the impact of varying silicon steel counts on the TPMLOA performance. The efficacy of this method is confirmed by comprehensive 3-D FEA and physical prototyping. The results show that the hybrid lamination meth-od can not only significantly improve electromagnetic thrust, but also greatly reduce the assembly difficulty and avoid assembly errors.

## 2. Topology OF TPMLOAs

### 2.1. Topology and operation principle of TPMLOAS

The mover of a LOA operates in a linear reciprocating manner. To increase the thrust density of the mover and reduce its mass, a PM structure for the mover is usually adopted. For linear reciprocating motion, the vertically oriented PM field must interact with a laterally varying magnetic field from the armature windings. Given the high reluctance of air, both fields experience significant attenuation. Enhancing the magnetic circuit with high-permeability materials is therefore essential to increase the field strength and improve force generation.

Fig 1 illustrates the topology of the TPMLOA, which adopts a dual-stator and single-mover structure to reduce the mover mass. The mover PM is composed of a multi-pole arrangement, with adjacent magnets having opposite magnetization directions. The support structure of the mover is made of titanium alloy material processed. Due to the fact that the closed-loop main magnetic circuit and the direction of motion of the rotor are located in the same plane, the outer stator adopts a C-shaped core structure, with concentrated windings placed inside it. The stator teeth are designed with semi-open slots to increase the overlapping area between the teeth and the PMs. Owing to the unique magnetic circuit configuration, the stator is typically manufactured from a single piece of soft magnetic material, which tends to result in high eddy current losses. In contrast, a stator made by laminating silicon steels can only be assembled using a circumferential stacking method. This approach not only leads to a tight inner structure and a loose outer structure, but also reduces the overall mechanical strength of the stator.

**Fig 1:**
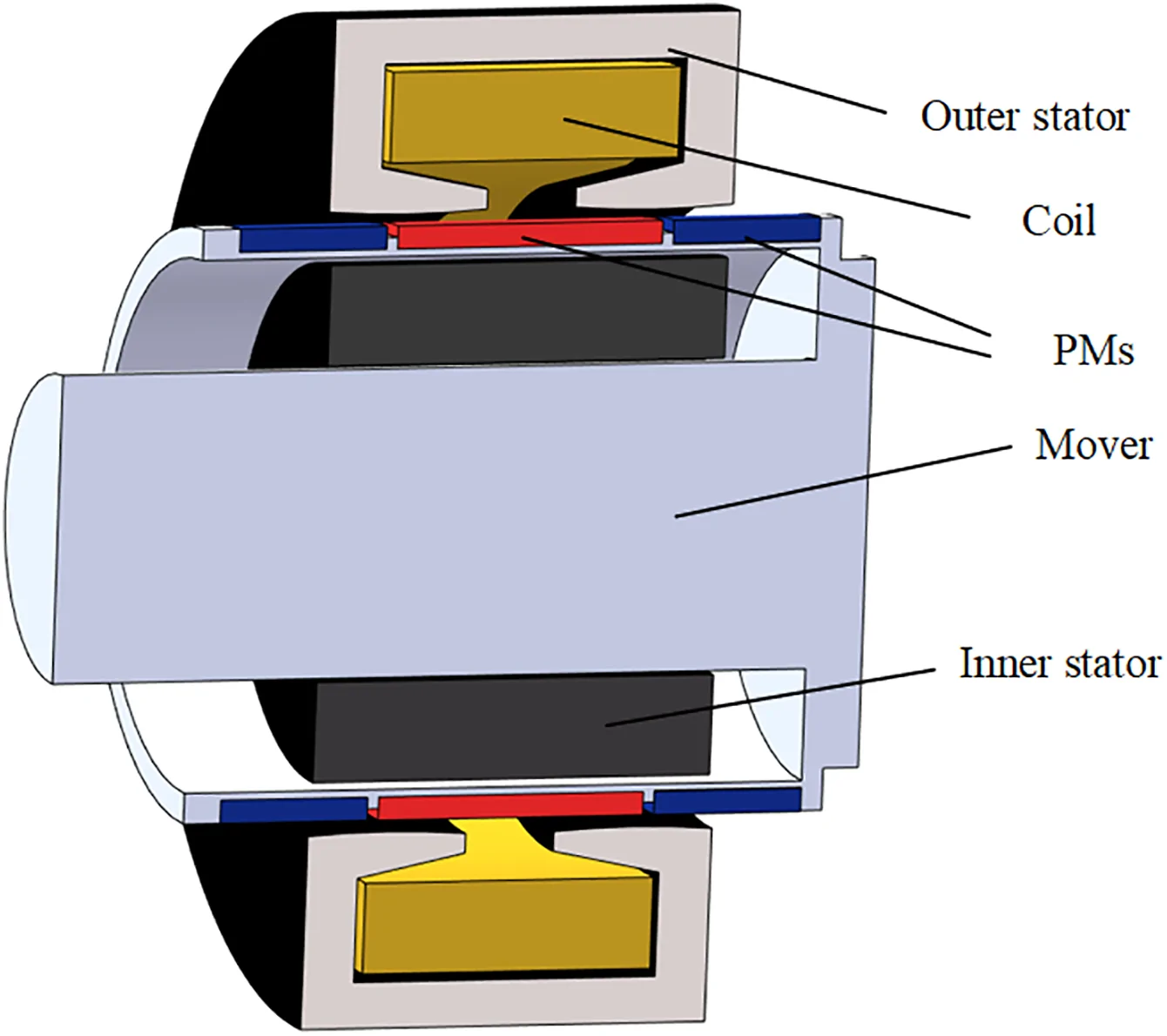
Topology of the TPMLOA.

### 2.2. Assembly technology

In order to reduce the difficulties in manufacturing and assembly processes, this paper proposes a hybrid lamination method in which silicon steels are embedded in soft magnetic materials. The hybrid lamination method combines the advantages of solid steel and silicon steels, thereby improving the stacking coefficient of the motor stator while avoiding a significant increase in eddy current losses.

Fig 2 illustrates a hybrid lamination scheme proposed in this paper. In Fig 2(a), the outer stator is composed of teeth and yoke. The outer stator teeth are made of a mixture of soft magnetic materials and silicon steels laminated together. Then a certain number of slots are opened on its surface from the inside out, so that the silicon steels can be tightly embedded. The depth of the slot should be less than the height of the soft magnetic material to ensure its integrity. Silicon steels and soft magnetic materials are fixed by locating rings. In order to save material costs, the outer stator yoke is assembled by radial lamination of silicon steels. The lamination method of the inner stator is shown in Fig 2(b). Silicon steels are embedded in the slots of the inner stator soft magnetic material and fixed by welding. The slotting depth of the inner stator soft magnetic material is less than the thickness of the stator to maintain structural integrity. Due to the correlation between the magnitude of eddy current losses and the magnetic cross-sectional area, it is possible to reduce eddy current losses by increasing the number of slots.

**Fig 2:**
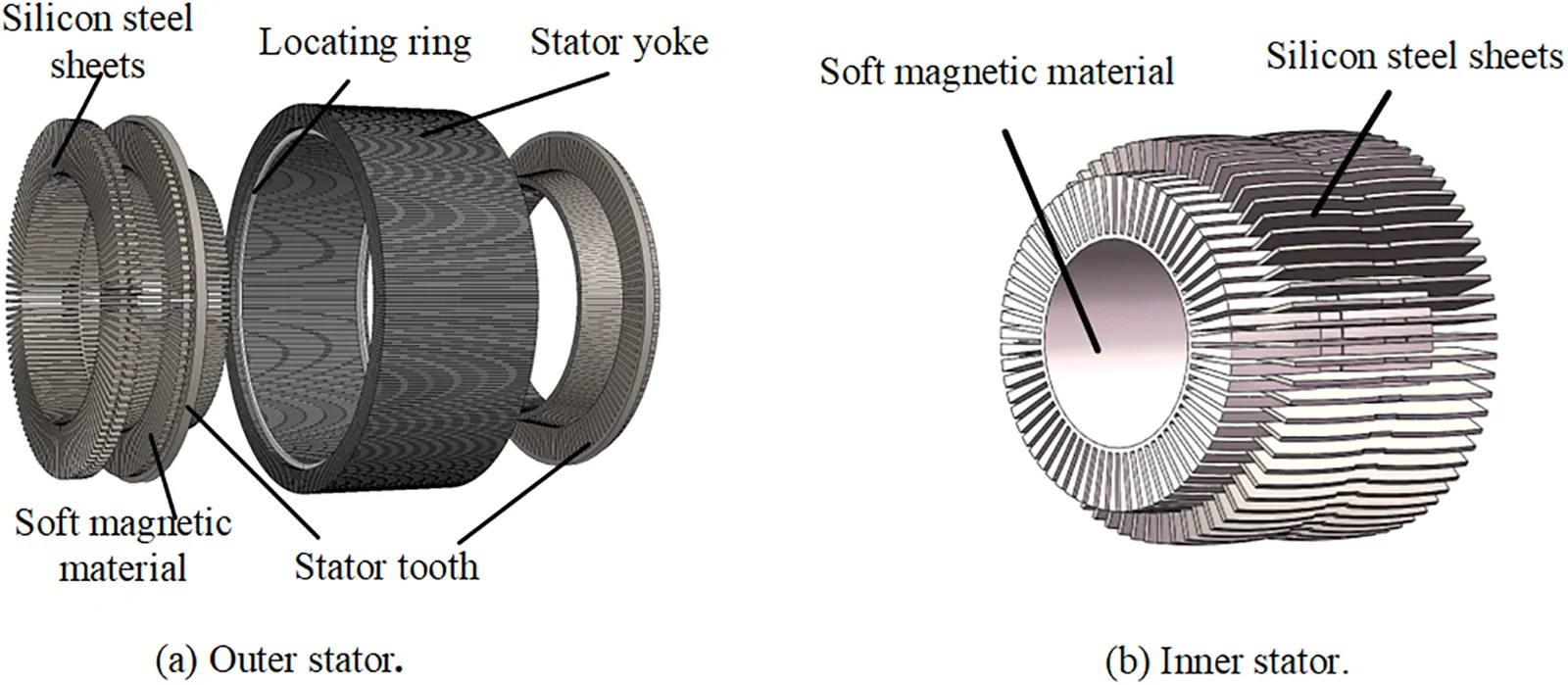
Hybrid lamination scheme of the TPMLOA.

The lamination coefficient of the TPMLOA using the hybrid lamination method has been significantly improved. Due to the large outer diameter and high ratio of inner and outer diameters of the stator yoke, the lamination coefficient can be approximately 1, and the overall stacking coefficient of the TPMLOA is also close to 1. In the inner stator, the cross-section of the component composed of soft magnetic material is fan-shaped. This structure results in eddy currents of different sizes generated by magnetic flux at different depths within the soft magnetic material. The area near the air gap is the largest, therefore more vortices will be generated. However, compared to PMLOA processed as a whole from soft magnetic materials, eddy current losses have been significantly reduced. Unlike the inner stator, the soft magnetic material in con-tact with the air gap at the outer stator teeth occupies a relatively small curved sur-face.

Unlike the inner stator, the soft magnetic material in contact with the air gap at the outer stator teeth occupies a relatively small curved surface. As the height of the outer stator increases, the upper surface of the fan-shaped structure becomes larger. During the slotting process, the number of slots can be increased to reduce the area occupied by the soft magnetic material. Due to the fact that the top of the outer stator teeth is machined from the entire soft magnetic structure, it results in significant eddy current losses. Therefore, during the manufacturing process, the outer stator yoke can be divided into multiple parts. Electrical insulation treatment can be carried out dur-ing the assembly process to further reduce eddy current losses.

## 3. Parameter calculation of hybrid lamination

There are significant differences in electromagnetic properties such as magnetic permeability and eddy current losses between silicon steel sheets and SMC materials. The hybrid lamination method proposed in this article requires mixing two materials together, so it is necessary to study the impact of different materials on the performance of the TPMLOA. The core of the hybrid lamination method is to embed silicon steel sheets into fan-shaped SMC materials. Fig 3 defines the key set parameters of the outer stator slot shape.

**Fig 3:**
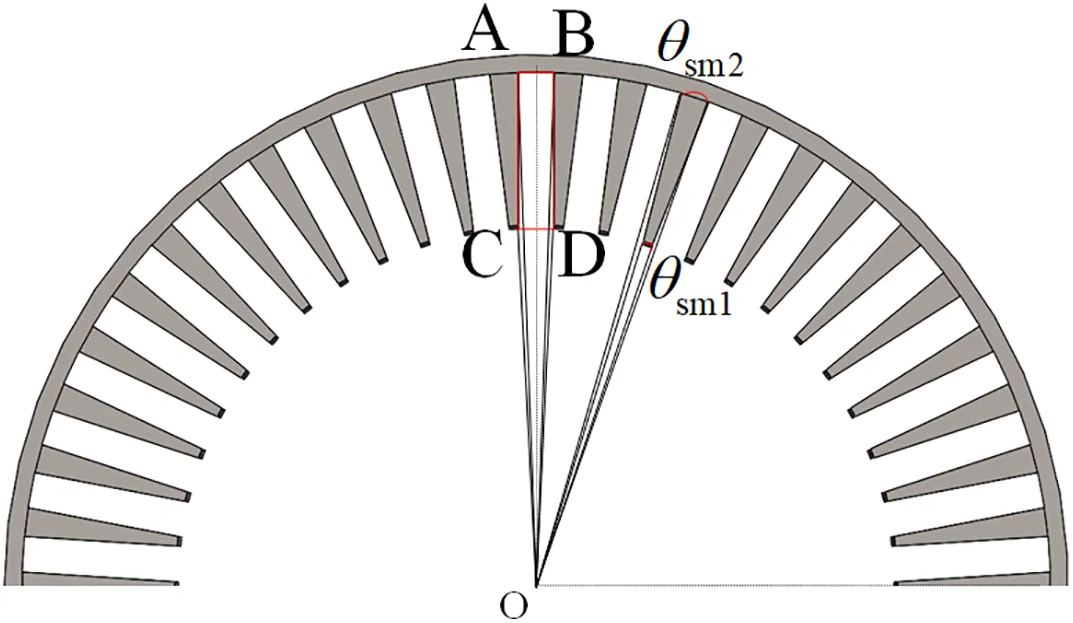
Schematic diagram of slotting for outer stator.

Taking the origin O as the center, the outer radius and inner radius of the outer stator are respectively denoted as $r_{\text{so}}$ and $r_{\text{si}}$. Assuming the number of silicon steel sheets embedded in each slot is $N_{\text{s}}$ and the thickness of a single silicon steel sheet is $h_{\text{s}}$, the physical width $W_{\text{slot}}$ of the slot is given.


$$\begin{array}{c} W_{\text{slot}}=N_{\text{s}}\cdot h_{\text{s}} \end{array}$$ (1)

This width corresponds to the chord lengths AB and CD. According to the geometric relationship, the central angles $\alpha_{\text{s}}$ and $\alpha_{\text{d}}$ corresponding to the slot top (outer diameter) and the slot bottom (inner diameter) are:


$$\begin{array}{c} \alpha_{\text{s}}=2arcsin\left(\frac{W_{\text{slot}}}{2r_{\text{so}}}\right) \end{array}$$ (2)

$$\begin{array}{c} \alpha_{\text{d}}=2\text{arcsin}\left(\frac{W_{\text{slot}}}{2r_{\text{si}}}\right) \end{array}$$ (3)

The effective depth $h_{\text{slot}}$ of the slot (i.e., the length of the line segment AC) is not simply a difference in radius and needs to be obtained by the Pythagorean theorem:


$$\begin{array}{c} h_{\text{slot}}=\sqrt{r_{\text{so}}^{\text{2}}-\frac{{W_{\text{slot}}}^{\text{2}}}{\text{4}}}-\sqrt{r_{\text{si}}^{\text{2}}-\frac{{W_{\text{slot}}}^{\text{2}}}{\text{4}}} \end{array}$$ (4)

To accommodate this depth, the actual cutting depth $h_{\text{cut}}$ required on SMC material is given.


$$\begin{array}{c} h_{\text{cut}}=r_{\text{so}}-\sqrt{r_{\text{si}}^{\text{2}}-\frac{{W_{\text{slot}}}^{\text{2}}}{\text{4}}} \end{array}$$ (5)

Thus, the cross-sectional area $A_{\text{slot}}$ of a single slot can be approximated as a rectangular area:


$$\begin{array}{c} A_{\text{slot}}\approx W_{\text{slot}}\cdot h_{\text{slot}} \end{array}$$ (6)

The entire outer stator is evenly divided into $N_{\text{slo}t}$, forming $N_{\text{slo}t}$ of identical fan-shaped SMC units. The lower arc angle $\theta_{\text{sm}1}$ and upper arc angle $\theta_{\text{sm2}}$ of each unit are:


$$\begin{array}{c} \theta_{\text{sm}1}=\frac{2\pi }{N_{\text{slo}t}}-\alpha_{\text{a}} \end{array}$$ (7)

$$\begin{array}{c} \theta_{\text{sm2}}=\frac{2\pi }{N_{\text{slo}t}}-\alpha_{\text{d}} \end{array}$$ (8)

The radial width $w_{\text{1}}$ and $w_{\text{2}}$ of the corresponding tooth root and tooth tip are key factors in evaluating mechanical strength.


$$\begin{array}{c} w_{\text{1}}=2r_{\text{so}}sin\left(\frac{\theta_{\text{sm2}}}{\text{2}}\right) \end{array}$$ (9)

$$\begin{array}{c} w_{\text{2}}=2r_{\text{si}}sin\left(\frac{\theta_{\text{sm1}}}{\text{2}}\right) \end{array}$$ (10)

The total area $A_{\text{SMC}}$ of a single sector SMC unit is the area of a circular ring sector minus the area of the slot:


$$\begin{array}{c} A_{\text{SMC}}=\frac{\text{1}}{\text{2}}\theta_{\text{sm}2}r_{\text{so}}^{\text{2}}-\frac{\text{1}}{\text{2}}\theta_{\text{sm1}}r_{\text{si}}^{\text{2}}-A_{\text{slot}} \end{array}$$ (11)

Therefore, in the entire cross-section of the outer stator, the total proportion of silicon steels $k_{\text{steel}}$ and SMC material $k_{\text{SMC}}$ can be calculated by the following formula:


$$\begin{array}{c} k_{\text{steel}}=\frac{N_{\text{slot}}\cdot A_{\text{slot}}}{A_{total}} \end{array}$$ (12)

$$\begin{array}{c} k_{\text{SMC}}=1-k_{\text{steel}} \end{array}$$ (13)

where $A_{\text{total}}$ is the total cross-sectional area of the stator core, which is equal to $\pi \left(r_{\text{so}}^{\text{2}}-r_{\text{si}}^{\text{2}}\right)$.

According to the lamination method, the lower arc angle of the fan-shaped soft magnetic material is constrained as follows.


$$\begin{array}{c} \theta_{\text{sm}1}>0 \end{array}$$ (14)

$$\begin{array}{c} \frac{2\pi }{N_{\text{slo}t}}-\alpha_{\text{d}}>0 \end{array}$$ (15)

Table 1 shows the relationship between the number of slots on the SMC material of the outer stator and the total quantity of silicon steels is obtained. As the number of silicon steels in a single slot increases from 1 to 10, the total quantity of silicon steels gradually increases, while the number of slots and the total area of SMC material gradually decrease.

**Table 1:** Relationship between the number of slots and silicon steels of outer stator.

Number of silicon steels per slot	Number of slots	Total number of silicon steels	Total area of SMC material (mm^2^)
*1*	251	251	3160
*2*	167	334	2440
*3*	125	375	2080
*4*	100	400	1860
*5*	83	415	1730
*6*	71	426	1630
*7*	62	434	1560
*8*	55	440	1510
*9*	50	450	1420
*10*	45	450	1420

## 4. Optimization design

To investigate the impact of different hybrid lamination schemes for the outer stator on the electromagnetic performance of the PMLOA motor, simulation analyses were conducted for various outer stator assembly methods while keeping the structures of other motor components unchanged. Fig 4 presents schematic diagrams of the outer stator structures with different hybrid lamination schemes. For the inner stator, a single silicon steel per slot lamination approach was adopted.

**Fig 4:**
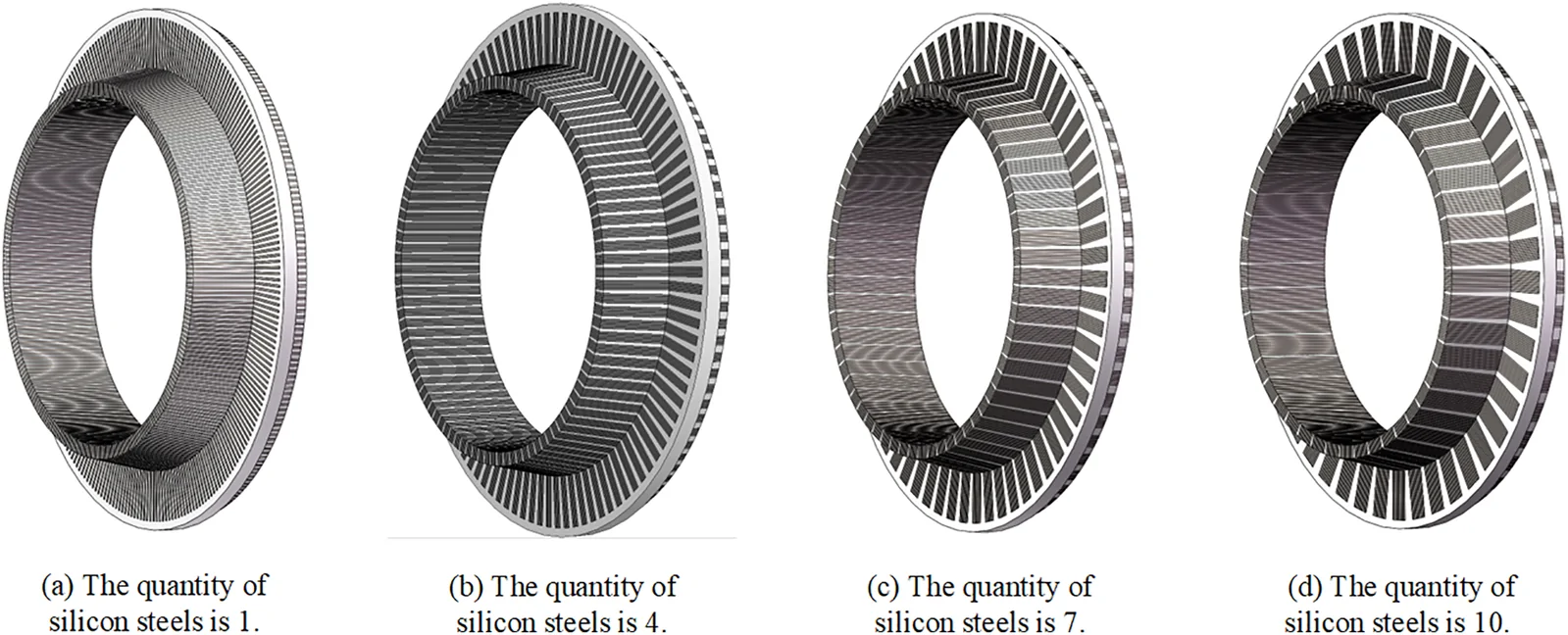
Schematic diagrams of the outer stator structures with different hybrid lamination schemes.

3D models of the outer stator are constructed and analyzed using Maxwell simulation software. The force coefficient of TPMLOA under no-load conditions was obtained through simulation while the rotor was running at a constant speed, as shown in Fig 5. As illustrated, the thrust coefficient of the TPMLOA exhibits a non-monotonic trend with the increasing number of silicon steels per slot in the outer stator. Initially, the higher permeability of silicon steels, compared to SMC material, lowers the overall magnetic reluctance and enhances the back electromotive force (back EMF). However, when the count exceeds 7 per slot, the enlarged air gap between the PM mover and silicon steels becomes the dominant factor. In addition, the small change in the total volume of silicon steels results in an overall increase in motor reluctance, leading to a decrease in output coefficient value.

**Fig 5:**
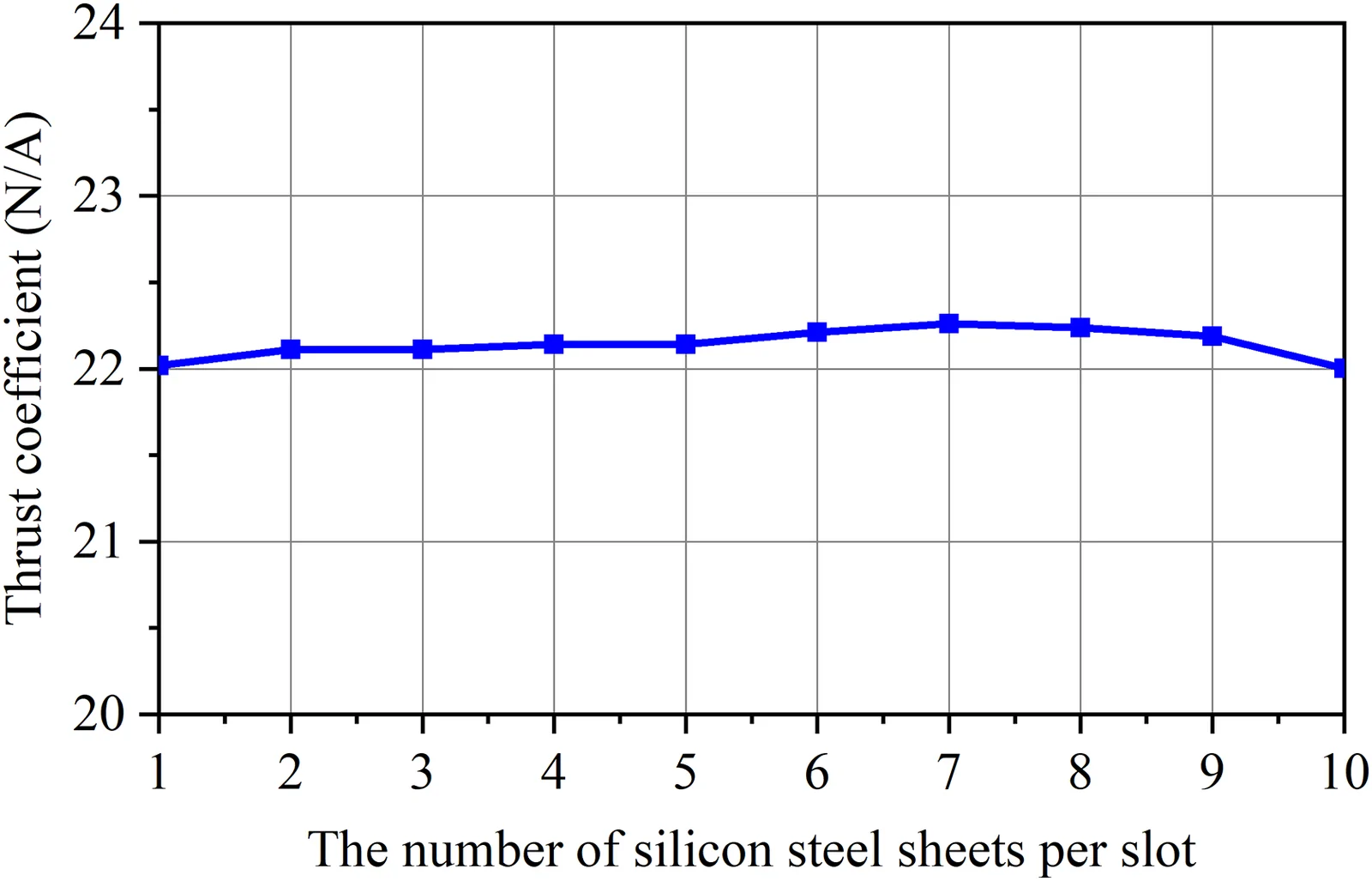
Trend of thrust coefficient variation of TPMLOA.

When the mover is at the maximum displacement position and the armature cur-rent is 10 A, the relationship curve between electromagnetic thrust and the number of silicon steels per slot is shown in Fig 6. The results indicate that the electromagnetic thrust at this time is only 150 N, and the thrust coefficient is significantly less than 22N/A. The main reason is that the armature current and PM magnetic field share the same magnetic circuit, leading to magnetic saturation. Due to this saturation, the overall variation in electromagnetic thrust with the number of silicon steels is minimal, remaining around 155 N. Notably, a significantly drop in thrust occurs specifically with 7 laminations per slot, which is directly related to the magnetic resistance characteristics of the TPMLOA main magnetic circuit.

**Fig 6:**
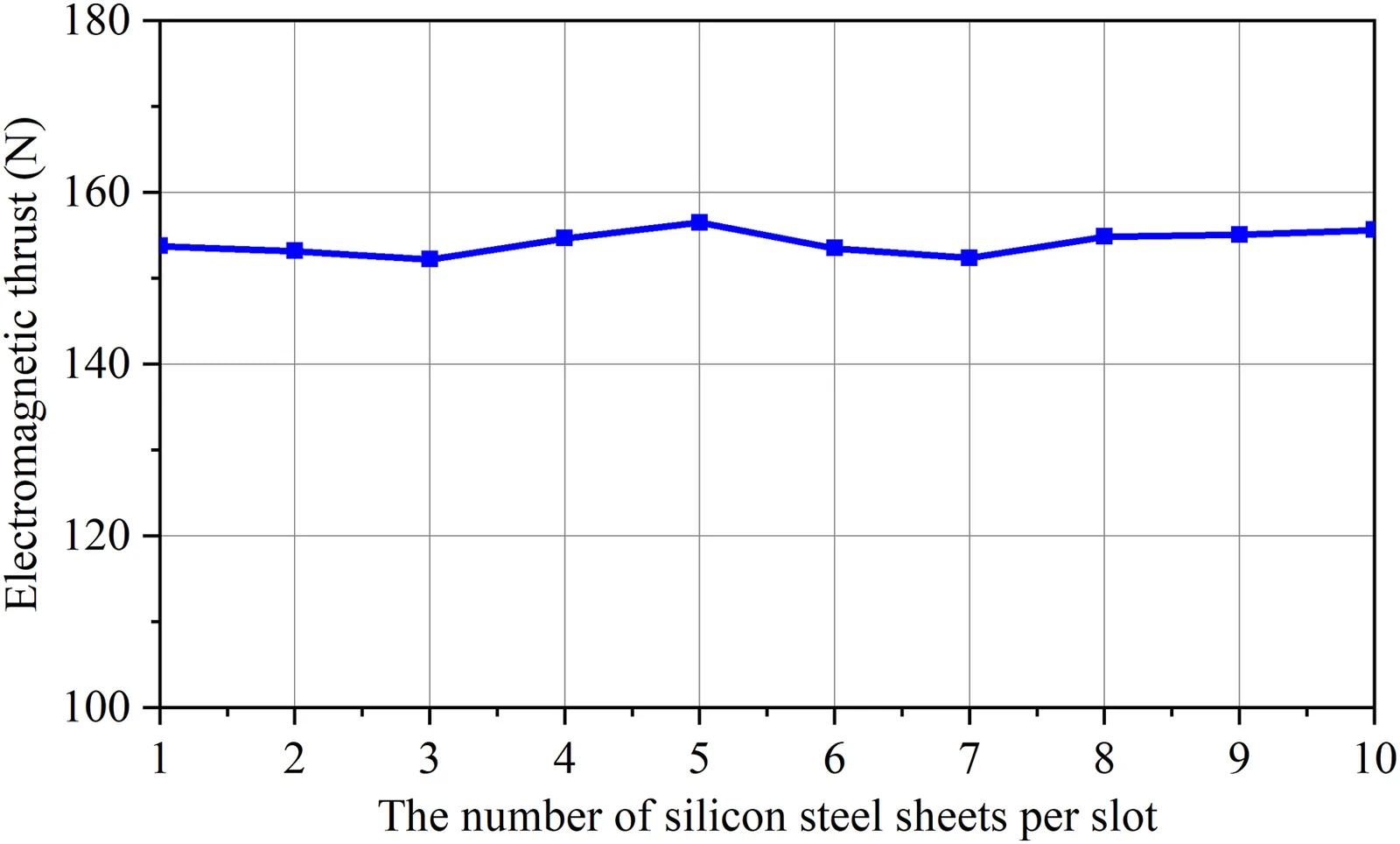
Trend of the electromagnetic thrust variation of TPMLOA.

Analyze the impact of hybrid stacking method on motor losses based on the iron core loss formula proposed by Berto et al [21].


$$\begin{array}{c} p_{\text{Fe}}=p_{\text{h}}+p_{\text{e}}+p_{\text{x}}=k_{\text{h}}f_{\text{c}}B_{\text{c}}^{\text{2}}+k_{\text{e}}f_{\text{c}}^{\text{2}}B_{\text{c}}^{\text{2}}+k_{\text{a}}f_{\text{c}}^{1.5}B_{\text{c}}^{1.5} \end{array}$$ (16)

where $p_{\text{h}}$ is hysteresis loss. $p_{\text{e}}$ is eddy current losses. $p_{\text{x}}$ is additional losses. $k_{\text{h}}$ represents hysteresis loss coefficient. $k_{\text{e}}$ represents eddy current loss coefficient, and $k_{\text{a}}$ represents additional loss coefficient. $B_{\text{c}}$ is the amplitude of the magnetic field density and $f_{\text{c}}$ is the frequency of the magnetic density waveform change.

Fig 7 illustrates the effect of the silicon steel count per slot on TPMLOA losses. Core loss shows a gradual increase with the rising count, while eddy current loss exhibits fluctuating changes. The main reason is that although the total amount of soft magnetic materials gradually decreases, the volume of a single fan-shaped soft magnetic material increases accordingly, resulting in a non-monotonic trend in eddy cur-rent losses. Specifically, when the silicon steel count per slot is 2, the total eddy current loss decreases compared to 1 sheet per slot, and then gradually increases with the in-crease of the silicon steel count. When the silicon steel count per slot is 9, there is a de-crease, mainly due to the fact that the width of the lower end of the outer stator fan-shaped soft magnetic material is 0.52 mm, which is smaller than the corresponding width when the silicon steel count is 8 and 10.

**Fig 7:**
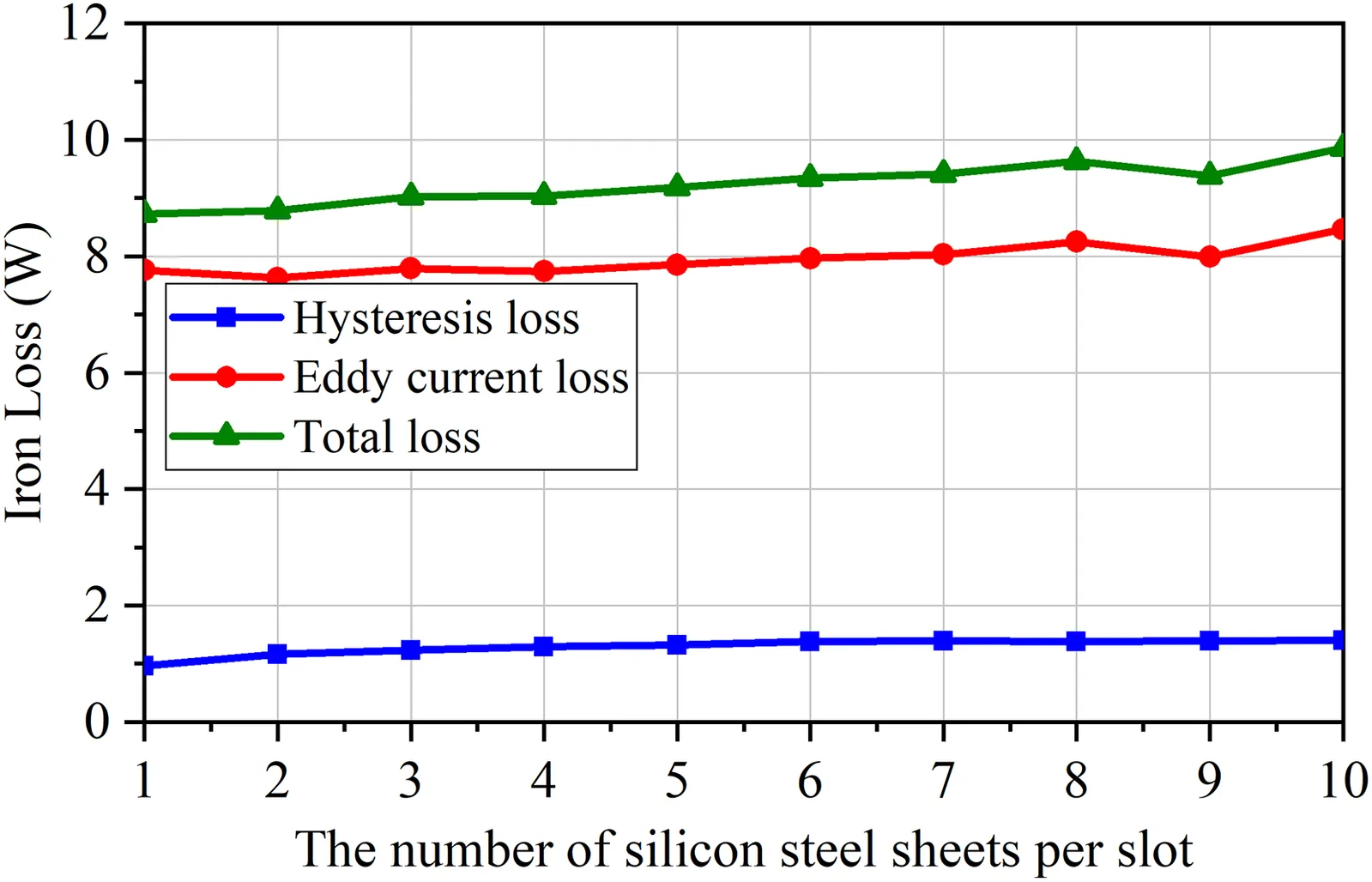
Effect of the number of silicon steels per slot on losses of TPMLOA.

Fig 8 shows the effect of the silicon steel count per slot on efficiency. It can be seen that the TPMLOA efficiency first rises and then declines with an increasing number of silicon steels per slot. When the silicon steel count per slot is 7, the efficiency of the TPMLOA reaches a maximum of 88.49%. The results indicate the silicon steel count per slot has a negligible impact on TPMLOA efficiency, with only a 0.19% difference between the maximum and minimum efficiency values. Consequently, to avoid increasing assembly complexity, the configuration with one silicon steel per slot in the outer stator was selected as the final prototype design.

**Fig 8:**
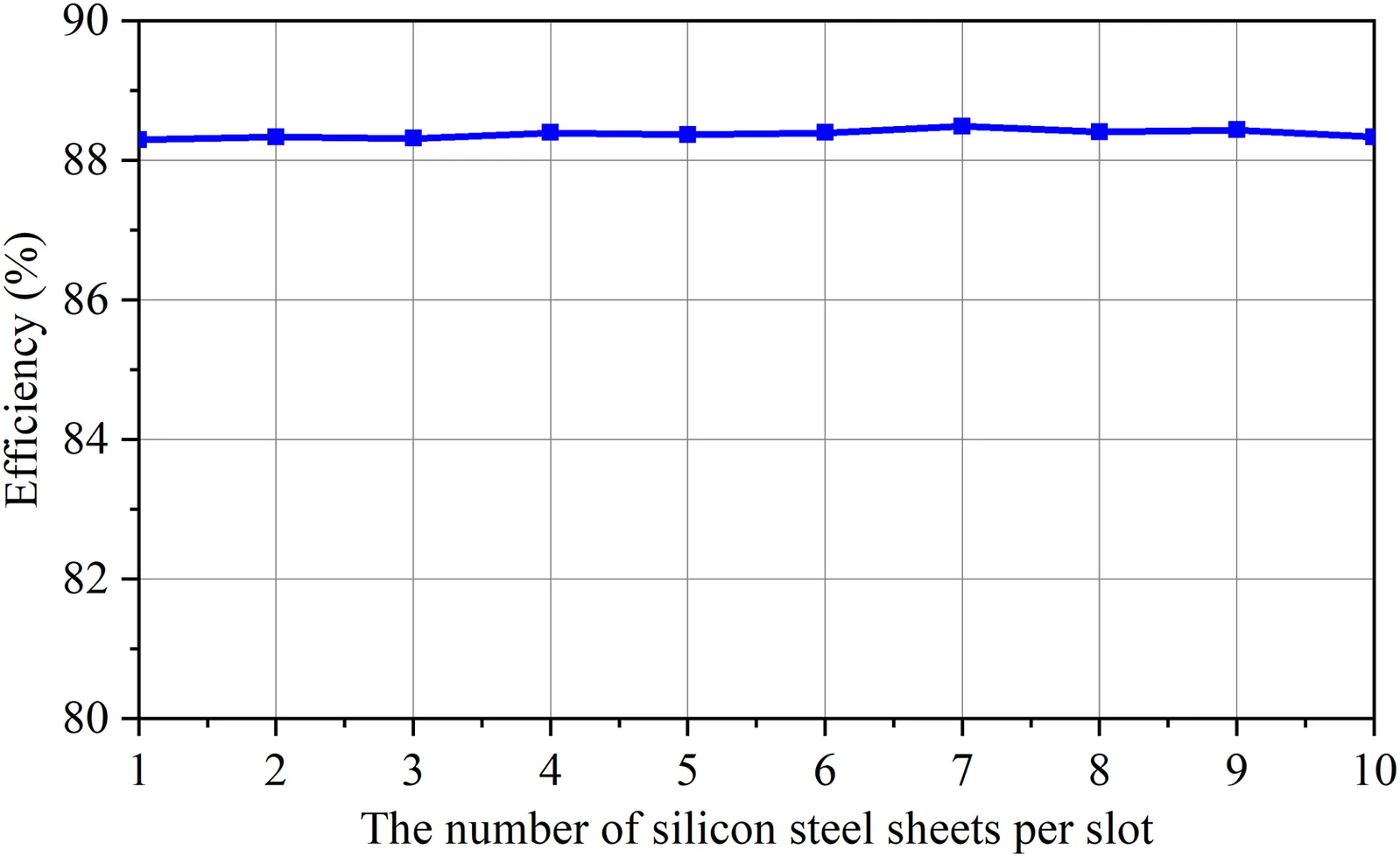
Influence of the number of silicon steels per slot on efficiency.

## 5. Simulation and experimental verification

### 5.1. Simulation comparison

Based on the preceding analysis of the hybrid lamination for the outer stator, a 3D structural model is created using finite element simulation software. The hybrid lamination stator employs two materials: silicon steel 50WW470 and SMC materials Somaloy 1000 3P. Additionally, two different lamination schemes were designed for the TPMLOA model, including traditional radial lamination and SMC materials Somaloy 700HR 3P integral processing. The characteristics of three materials are shown in Table 2. Among them, Somaloy 700HP 3P material is currently a cost-effective SMC material used for overall motor processing. But its structural strength is not suitable for the slotting treatment in the design scheme. Therefore, a Somaloy 1000 3P material with relatively poor electromagnetic performance but high structural strength was chosen. The impacts of these three lamination schemes on TPMLOA performance are compared through 3D FEA. Fig 9 shows the simulation 3D models of TPMLOAs with three different assembly methods. Due to the large number of silicon steels contained in the TPMLOA structure, the number of mesh elements in the model mesh is relatively large. In order to reduce simulation time, one twelfth of the model is used in the simulation.

**Table 2:** Electromagnetic properties of silicon steel sheets and SMC materials.

Soft magnetic material	Density g/cm^3^	Resistivity μΩ·𝐦	Magnetic induction intensity B @ 10000A/m	Maximum permeability $\mu_{max}$	Magnetic core loss W/kg @1.0T 50 Hz
**Somaloy 700HR 3P**	7.52	600	1.57	770	5.10
**Somaloy 1000 3P**	7.56	70	1.63	900	5.20
**50WW470**	7.70	4.6	1.82	2200	1.12

**Fig 9:**
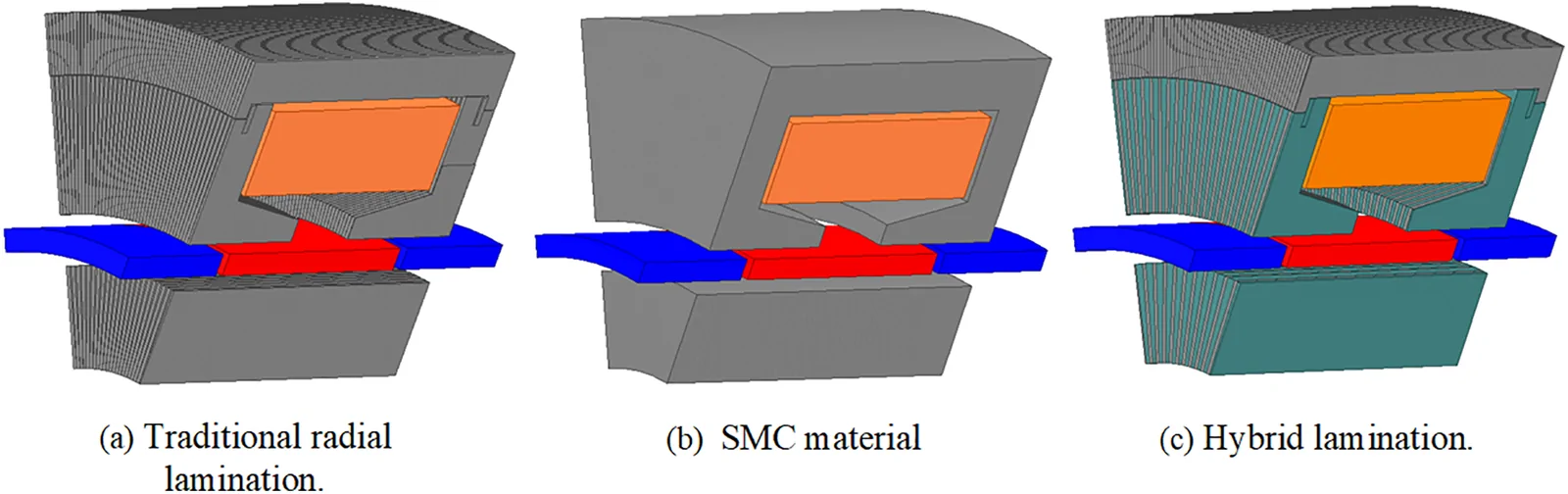
3D models of TPM LOAs with three different assembly methods.

The magnetic flux density in the magnetic circuit is a key indicator for evaluating the utilization efficiency of materials. A higher magnetic flux density can easily lead to magnetic saturation under load conditions, while a lower magnetic flux density can limit the power density of the motor. Therefore, the reasonable selection of magnetic flux density is of great significance for optimizing material usage, balancing performance and cost. To analyze the magnetic field distribution characteristics of different types of TPMLOAs, simulation studies are conducted on their air gap magnetic flux density under no-load operating conditions. To further observe the magnetic density distribution inside the silicon steels of the stator teeth, an XY plane section is taken in the middle of the stator teeth, as shown in Fig 10.

**Fig 10:**
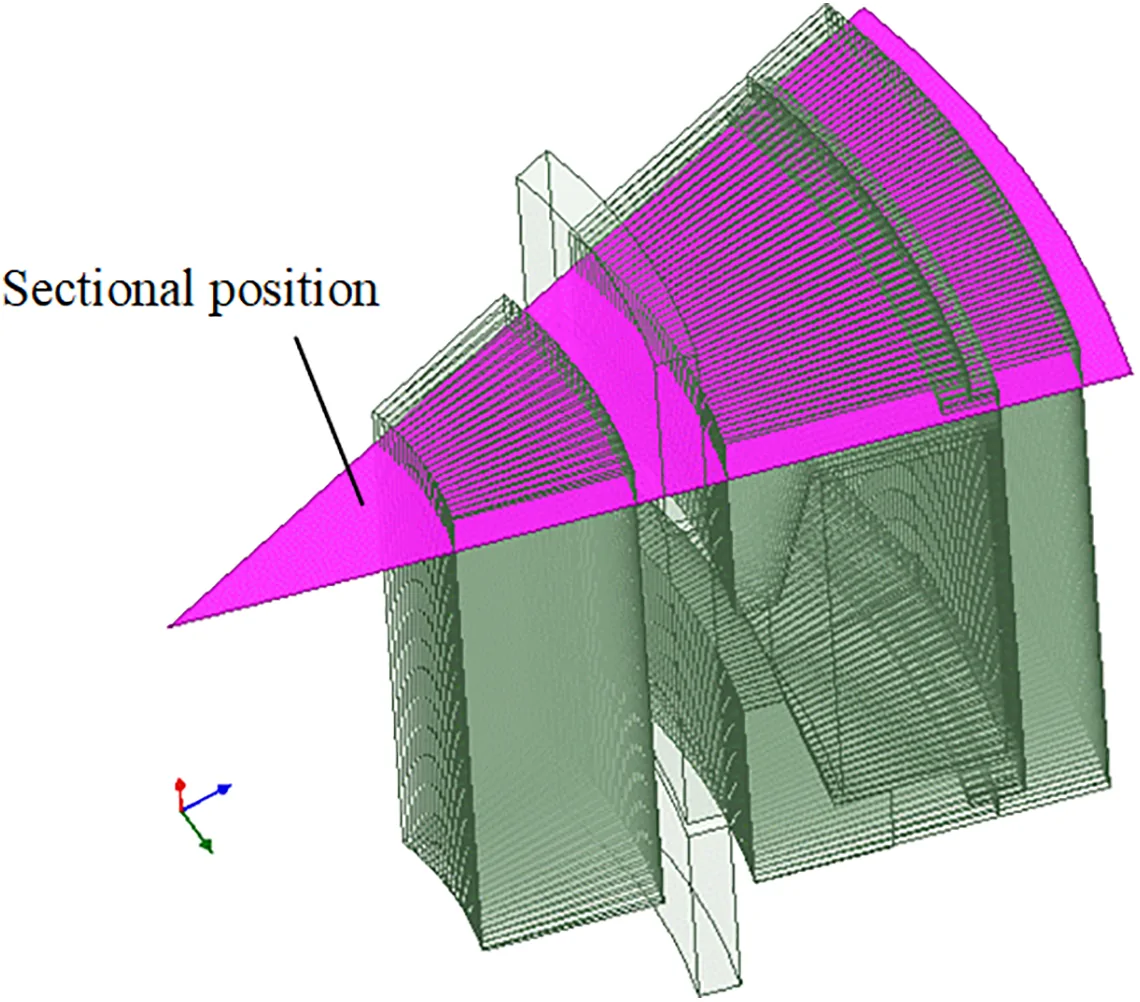
Cross-sectional position for magnetic flux density of TPMLOA.

Fig 11 shows the influence of three lamination methods on the magnetic flux density of a TPMLOA. By comparing and analyzing the magnetic flux density maps of three types of TPMLOAs at the same position, it can be concluded that in the scheme using only the circumferential lamination of silicon steels, the amplitude of the magnetic flux density inside the silicon steels is relatively high. This is mainly due to the air gap between the silicon steels, which makes the main magnetic circuit more prone to saturation after the load current is applied. The stator structure made of integral SMC has a relatively uniform magnetic flux density distribution and a lower overall magnetic flux density level. In the hybrid lamination scheme proposed in this paper, the magnetic flux density in the silicon steel area is about 1.3 T, while the SMC part exhibits a low and uniform magnetic density distribution. This structure can effectively suppress the magnetic saturation phenomenon during load operation.

**Fig 11:**
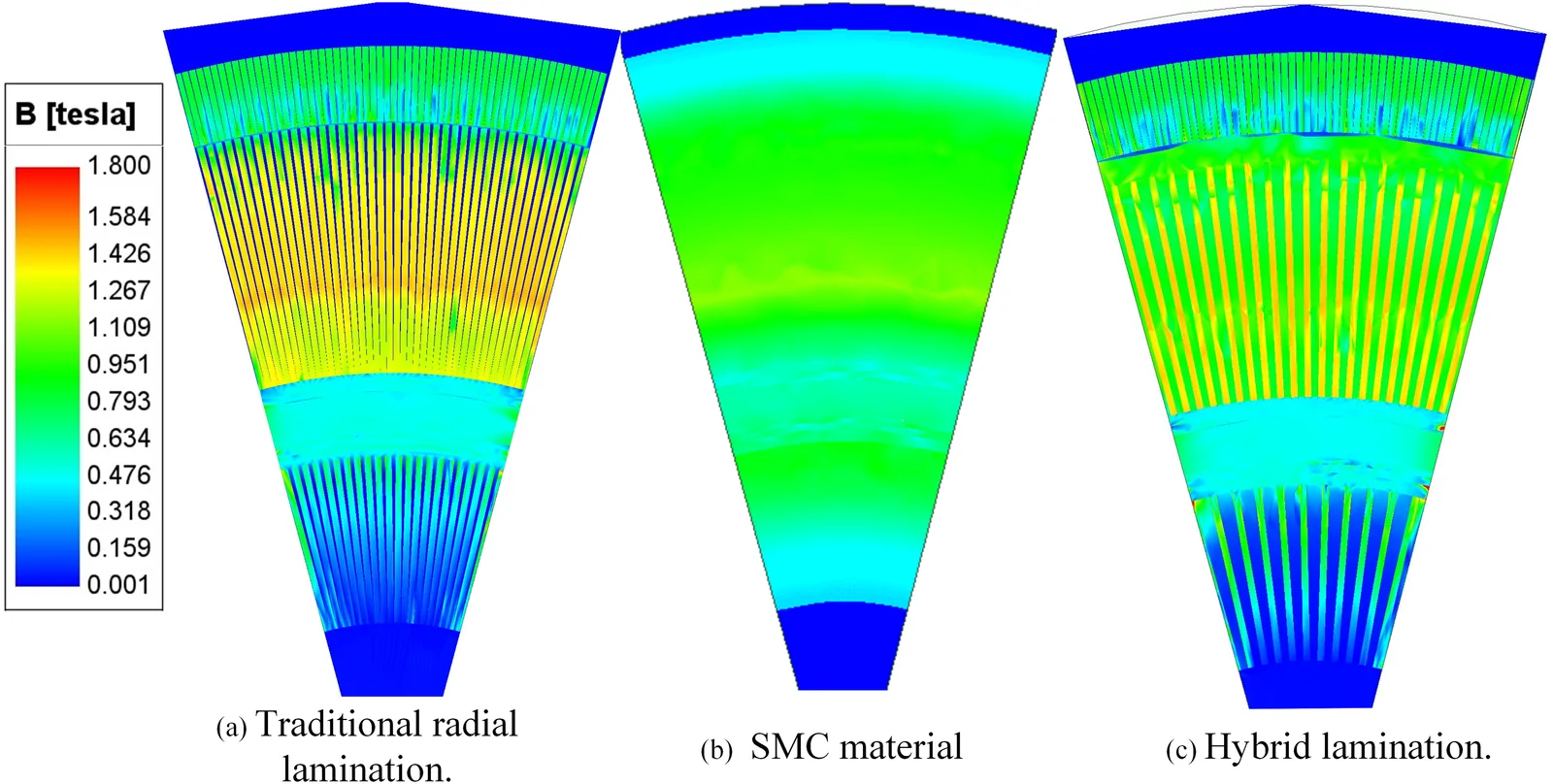
Magnetic field density distribution map of TPMLOA.

When the load current is 20A and the mover is at its maximum position, the magnetic density diagram of TPMLOAs using different lamination methods is shown in Fig 12. The armature and the PM magnetic field share the same magnetic circuit, and the magnetic circuit is in a superimposed state, so the saturation of the magnetic circuit is most obvious. In Fig 12(a), the saturation of magnetic density is relatively severe, and the magnetic field density in the outer stator silicon steels basically exceeds 1.8T. The TPMLOA made of SMC materials also has a certain degree of saturation, as shown in Fig 12(b), but the saturation degree is significantly smaller compared to the traditional radial lamination. This is mainly due to the SMC materials scheme being able to increase the magnetic conductivity area. The saturation degree of the stator assembled by hybrid lamination method is effectively improved as shown in Fig 12(c). The maximum magnetic field density in the inner stator is only 1.5T, and only a certain degree of magnetic field saturation occurs in the silicon steels of the outer stator.

**Fig 12:**
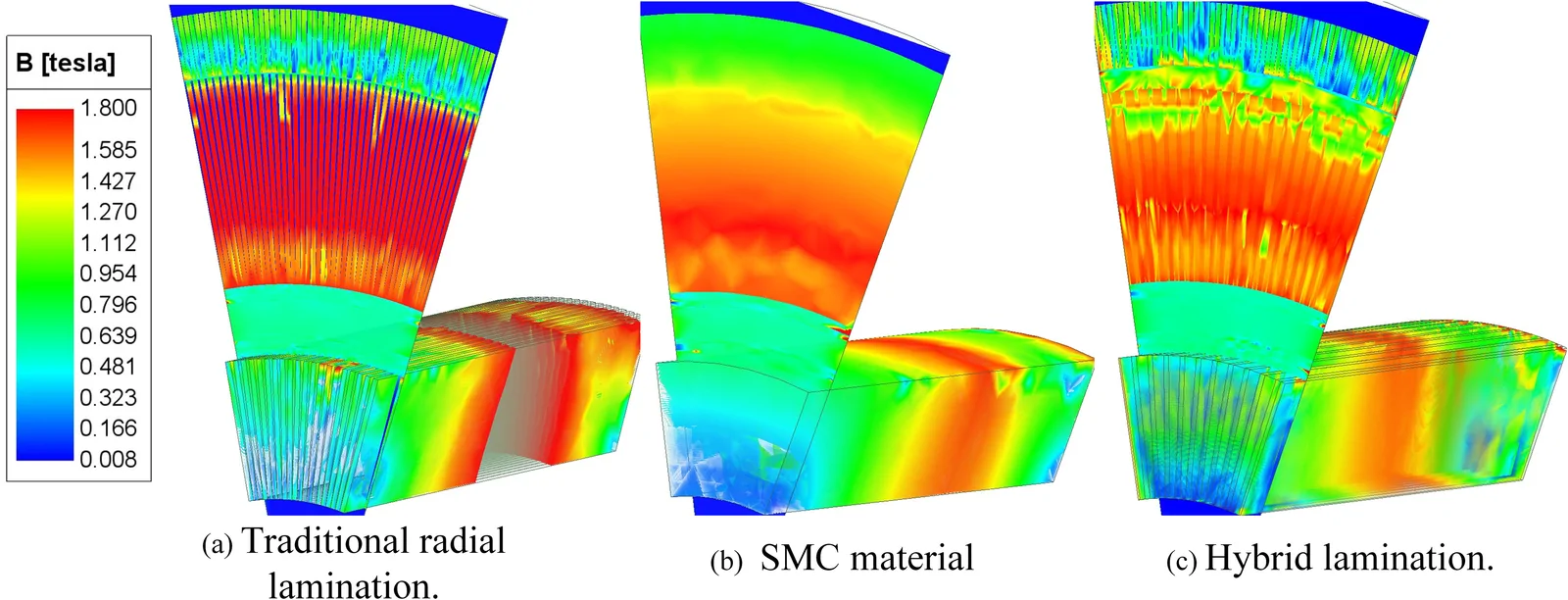
Comparison of magnetic density under load state of the TPMLOAs with different lamination modes.

The waveform comparison of the back EMF of the TPMLOAs is shown in Fig 13. At this time, the mover of TPMLOA is operating in a sinusoidal state with a frequency set to 40 Hz, a current of 0A, and an effective stroke of ± 8 mm. The amplitude of the back EMF waveform generated by traditional radial lamination and hybrid lamination methods of silicon steels is 49.4V. However, the back EMF of TPMLOA cast with SMC materials is significantly smaller than the first two methods. This is mainly due to the fact that SMC materials reduce their magnetic permeability in order to in-crease their electrical resistivity.

**Fig 13:**
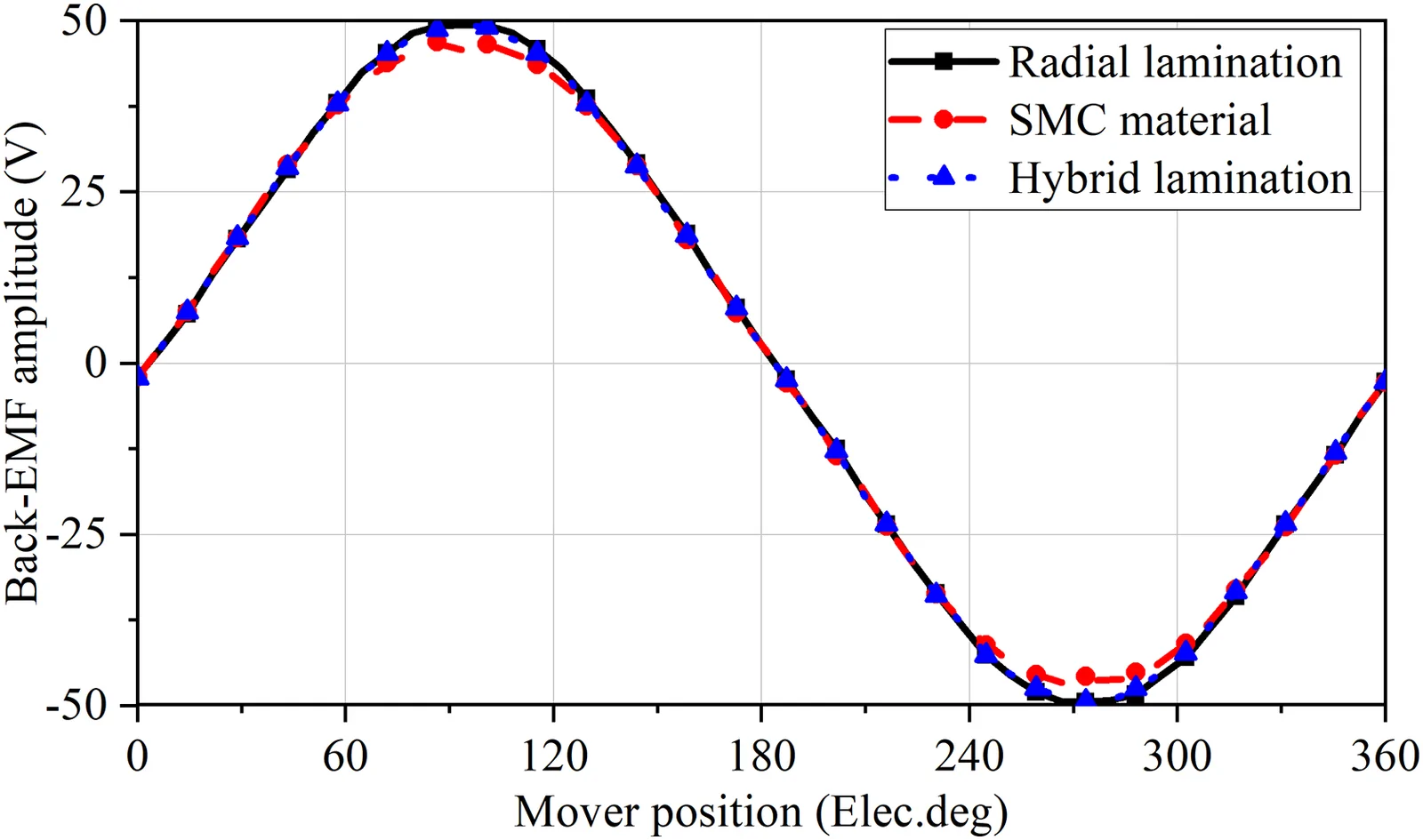
Comparison of back EMF of the TPMLOAs with different lamination modes.

When a 20 A DC current is applied to the armature winding, simulation analysis is conducted on the electromagnetic force at different positions of the mover. The comparison results of electromagnetic thrust of three TPMLOAs at different positions are shown in Fig 14. As shown in the figure, when the mover is in the negative position (i.e., the direction of the armature current magnetic field is opposite to that of the permanent magnet magnetic field), the electromagnetic force of the silicon steel laminating and the hybrid laminating of TPMLOA is basically the same. As the position of the mover changes, the direction of the current magnetic field and the PM magnetic field become the same, and the magnetic circuit gradually enters saturation, resulting in a gradual decrease in the amplitude of electromagnetic thrust compared to the symmetrical position. Among them, the electromagnetic force by the silicon steel laminating motor decreases more significantly, significantly lower than the hybrid laminating scheme. This is mainly due to the lower lamination coefficient when the inner and outer stators are laminated with silicon steels, which exacerbates the saturation degree of the magnetic circuit. The TPMLOA made of integral SMC materials die-casting has an electromagnetic force equivalent to that of the hybrid lamination scheme at the maximum displacement, but its average electromagnetic force throughout the entire mover stroke is significantly lower than the first two structures.

**Fig 14:**
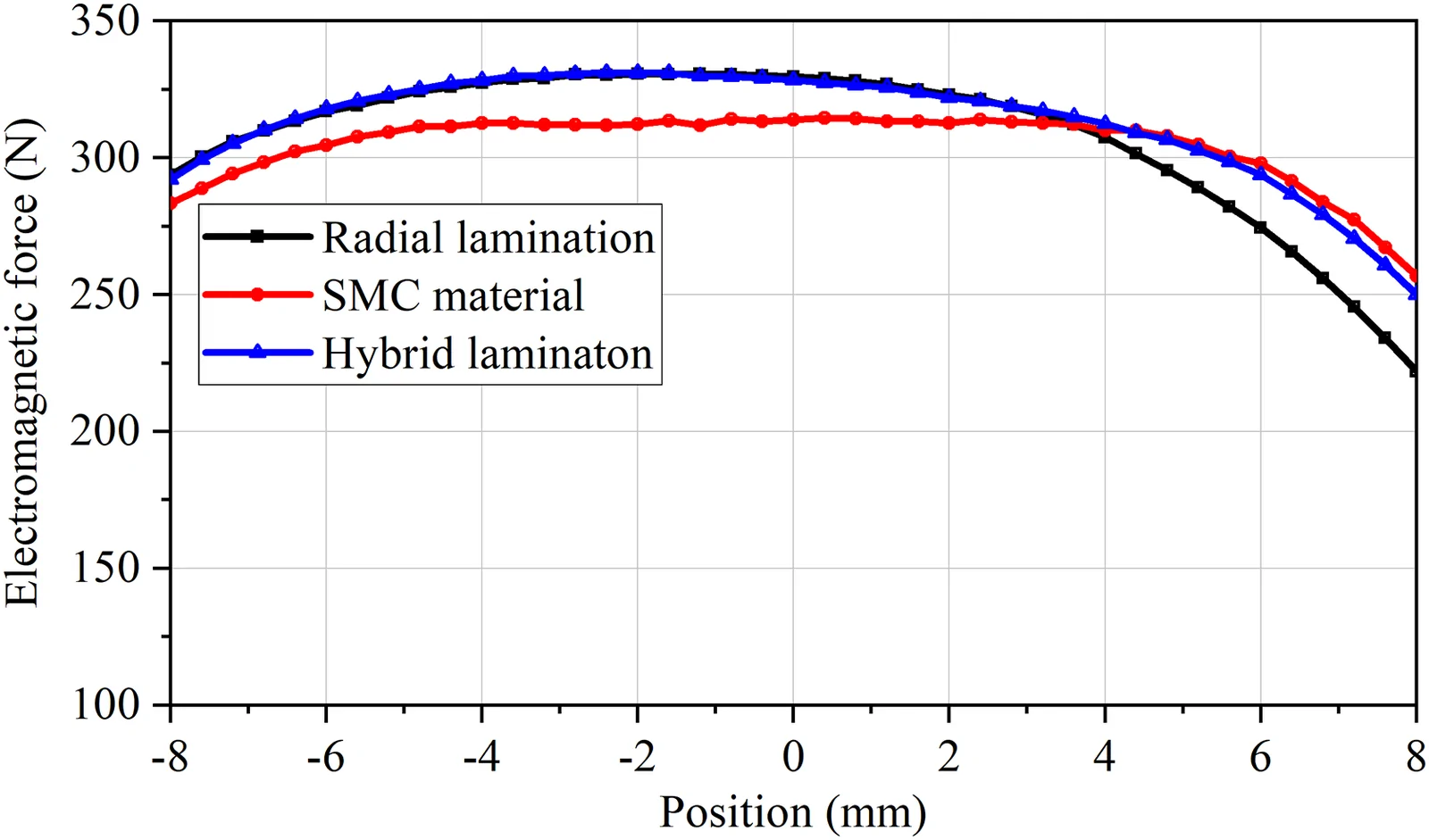
Comparison of electromagnetic force of the TPMLOAs with different lamination modes.

When the TPMLOA operates at a sinusoidal speed, with a working frequency of 40 Hz, an effective stroke of ± 8 mm, and an AC amplitude of 20A, the iron core loss and eddy current loss generated by the motors with three lamination modes are simulated and obtained as shown in Table 3. Due to the small number of silicon steels in the hybrid lamination, the total iron core loss is lower than that of TPMLOAs that only use silicon steels for lamination. The maximum eddy current loss generated by the TPM-LOA using hybrid lamination method is 22.7W, which is twice that of traditional radial lamination (11.14 W) and about 38% higher than the design using only SMC (16.47W). The reason for the significant increase in eddy current losses is that the hybrid stator adopts a continuous, non-segmented solid Somaloy 1000 3P SMC material outer gear ring, forming a large closed conductive ring perpendicular to the AC magnetic flux. Unlike traditional silicon steel laminates, they have interlayer insulation and inherent eddy current suppression capabilities, while solid SMC materials retain continuous conductive paths. At the same time, in order to meet the structural strength requirements for stator slotting, the hybrid laminated stator adopts Somaloy 1000 3P with slightly poorer electromagnetic performance. Its conductivity is much lower than that of Somaloy 700HR 3P, but its material cost is lower than the latter. Despite this penalty, the thrust benefit significantly outweighs the loss cost for compressor applications. Compressor TPMLOAs operate under heavy load and require peak thrust exactly at the maximum mover displacement, where the hybrid design delivers a 26% thrust improvement and suppresses severe magnetic saturation. Compressors spend most of their cycle in this high-load, maximum-displacement region, making the hybrid strategy’s targeted thrust gain far more impactful than the moderate increase in total loss. Meanwhile, in the later stage, the eddy current losses can be reduced by using soft magnetic materials with better electromagnetic characteristics or by dividing the outer stator tooth ring into blocks.

**Table 3:** Comparison of iron core losses and eddy current losses generated by three TPMLOAs.

	Traditional radial lamination.	SMC material	Hybrid lamination.
**Iron core losses (W)**	4.55	8.59	3.16
**Eddy current losses (W)**	6.59	7.88	19.5
**Total loss (W)**	11.1	16.4	22.7

### 5.2. Experimental verification

A TPMLOA prototype is developed based on design parameters to verify the feasibility of the proposed hybrid stacking method in improving motor performance. In addition, a corresponding experimental testing platform has been established to systematically compare and verify the actual measurement results with the aforementioned simulation calculation results. Fig 15 displays the physical appearance of the TPMLOA prototype and the overall testing platform it is located on. Specifically, it can be clearly observed from Fig 15(a) and (b) that the core innovation of this proto-type lies in the use of a hybrid configuration of silicon steels and SMC materials on its outer stator teeth. The testing platform, as shown in Fig 15(c), integrates driving, measurement, and loading functions. The core equipment includes an AC power supply for driving, a power analyzer for measuring electrical parameters, a digital oscilloscope for displaying waveforms, a TPMLOA prototype, a load LOA for simulating loads, and a high-precision tension sensor for measuring thrust. Through standardized connections, each device ensures the synchronization and accuracy of data collection, providing a reliable foundation for performance comparison.

**Fig 15:**
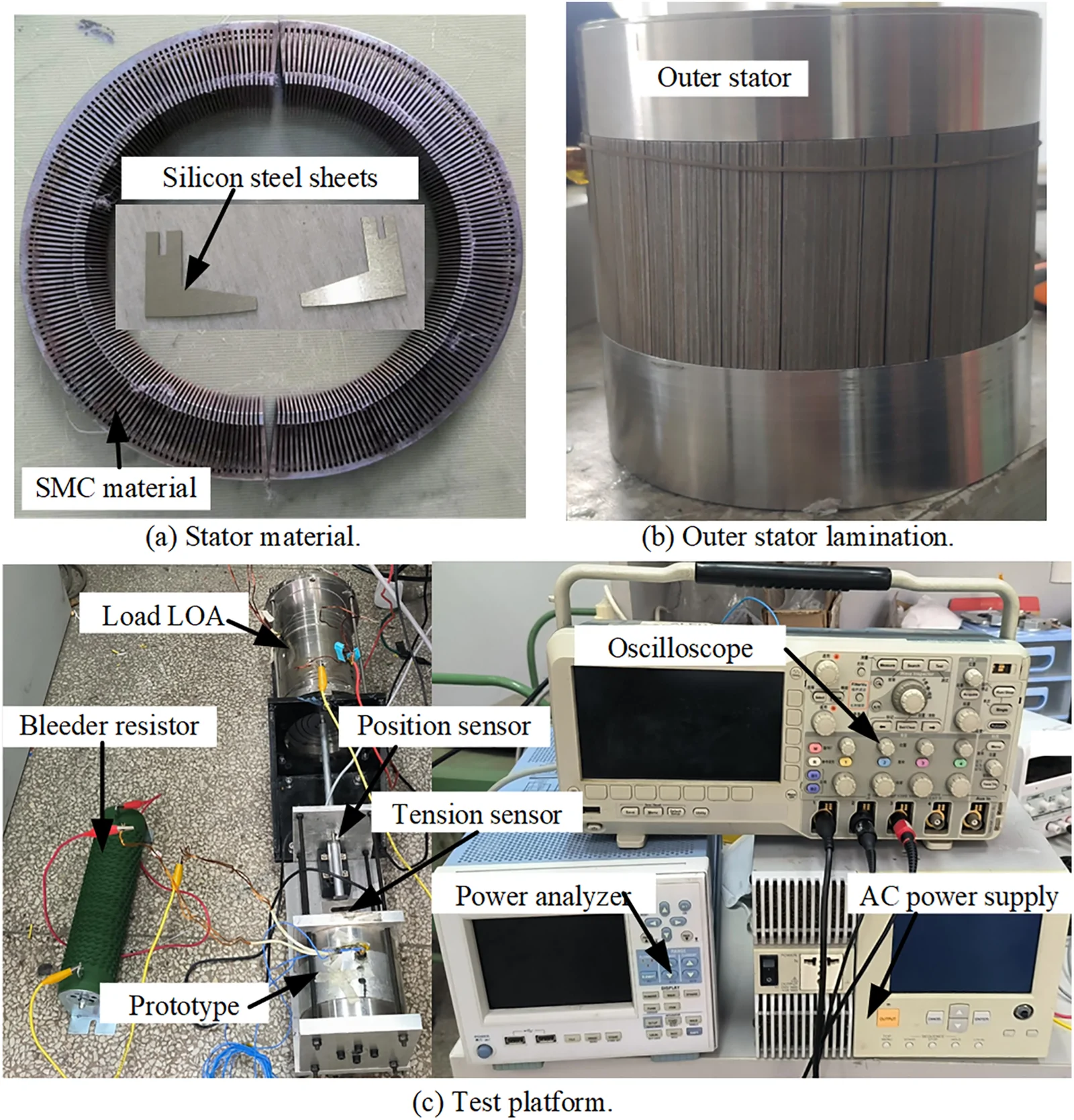
Prototype and test platform of the TPMLOA.

The comparison results of the back-EMF, with a frequency of 20 Hz, between 3D FEA and the experimental are depicted in Fig 16(a). The maximum value recorded in the back-EMF experiment is 18.5V, showing a 10% decrease when compared to the results of the 3-D FEA. This error is attributed to the machining tolerances of the stator core, small air gap misalignment, and changes in the properties of permanent magnets and silicon steel materials. It is worth noting that for customized tubular permanent magnet linear oscillation actuators, especially those with novel hybrid laminated structures, a simulation experiment difference of 8% −15% is typical [19]. Therefore, the observed 10% difference is within the expected engineering range, verifying the consistency between simulation and experiment. Meanwhile, due to assembly accuracy errors during the construction of the testing platform, the waveform of the back electromotive force obtained from the test is severely distorted. The assembly accuracy of the prototype itself, including the concentricity of the stator and rotor, as well as the roundness of the stator block, has a significant impact on the waveform of the back electromotive force. Fig 16(b) shows the comparative results of electromagnetic forces. It demonstrates that the trend of electromagnetic force variation obtained from the experiment is essentially consistent with the results of the 3D FEA. Since the 3D FEA did not account for machining, assembly errors, the actual test results are lower than the simulated results.

**Fig 16:**
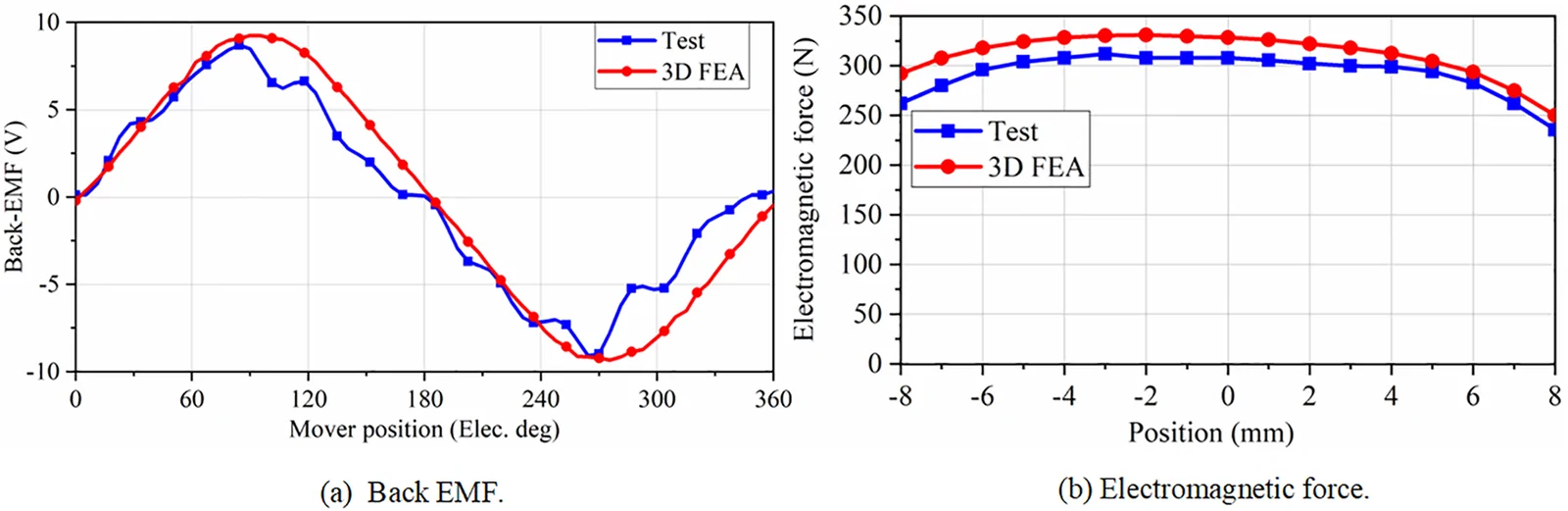
Comparison of tested and 3D simulation results.

The efficiency characteristic with frequency variation of the prototype is tested by the test platform. It can be seen from Fig 17 that the efficiency initially increases and then decreases as the frequency rises. When the low frequency is less than 30 Hz, copper losses inside the motor dominate. As the actuator approaches its resonant frequency, efficiency increases with increasing frequency, and the thrust to power ratio reaches its optimum. When the frequency is greater than 30 Hz, the eddy current loss in the continuous SMC material of the outer stator rapidly increases, resulting in an increase in the total loss of the motor and a sharp decrease in overall efficiency.

**Fig 17:**
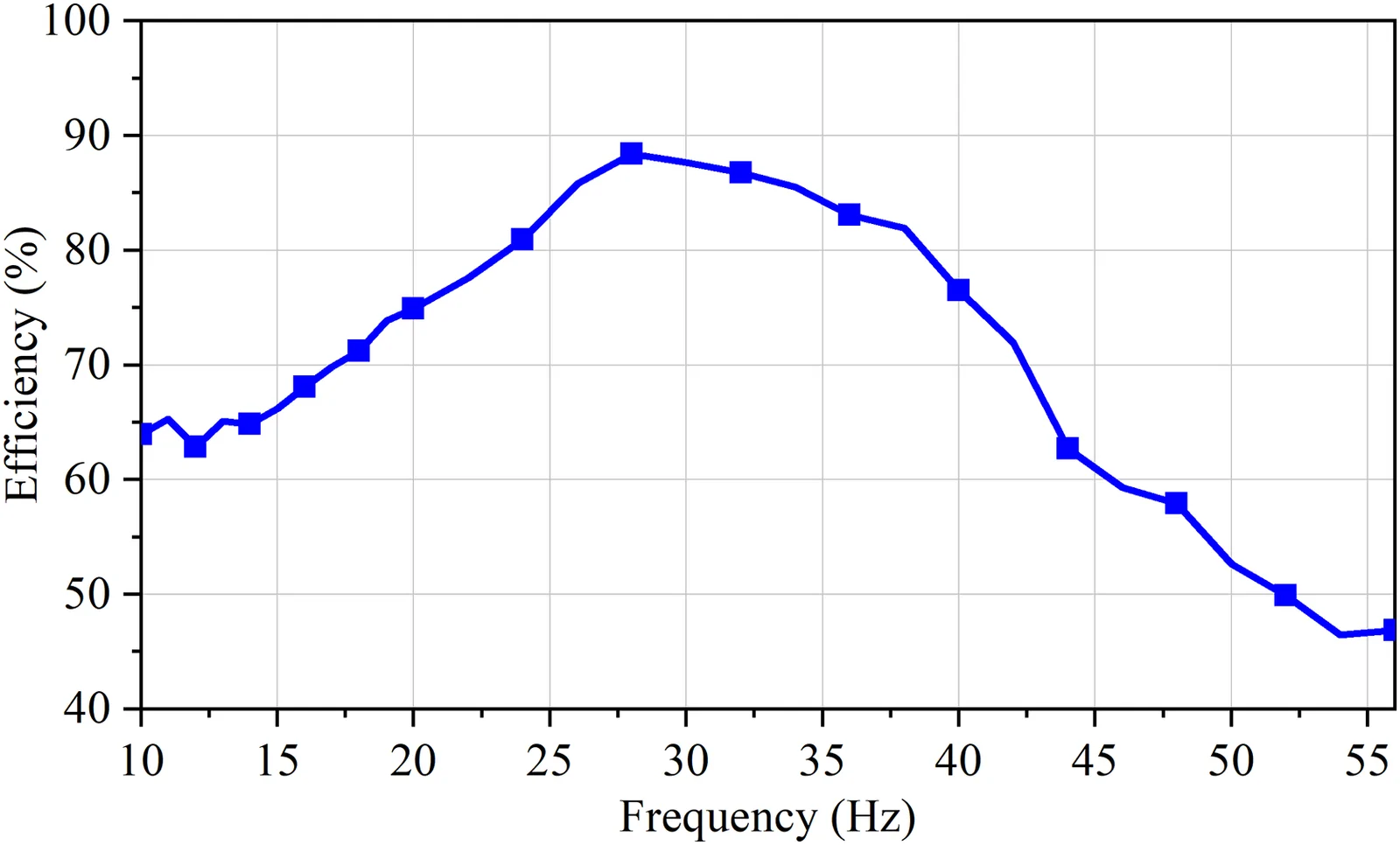
Efficiency waveform with frequency variation of the prototype.

The temperature curve of the prototype under load at a current of 20A is shown in Fig 18, and the experimental environment temperature is 27 °C. As shown in the figure, the highest temperature of the winding of the motor stabilizes at 105 °C after running for 2 hours, with a temperature rise of 78 °C. The surface temperatures of the motor stator teeth, stator yoke, and casing are 93.5 °C, 84.5 °C, and 75.6 °C, respectively. The temperature rise experiment shows that the motor can operate stably under rated conditions.

**Fig 18:**
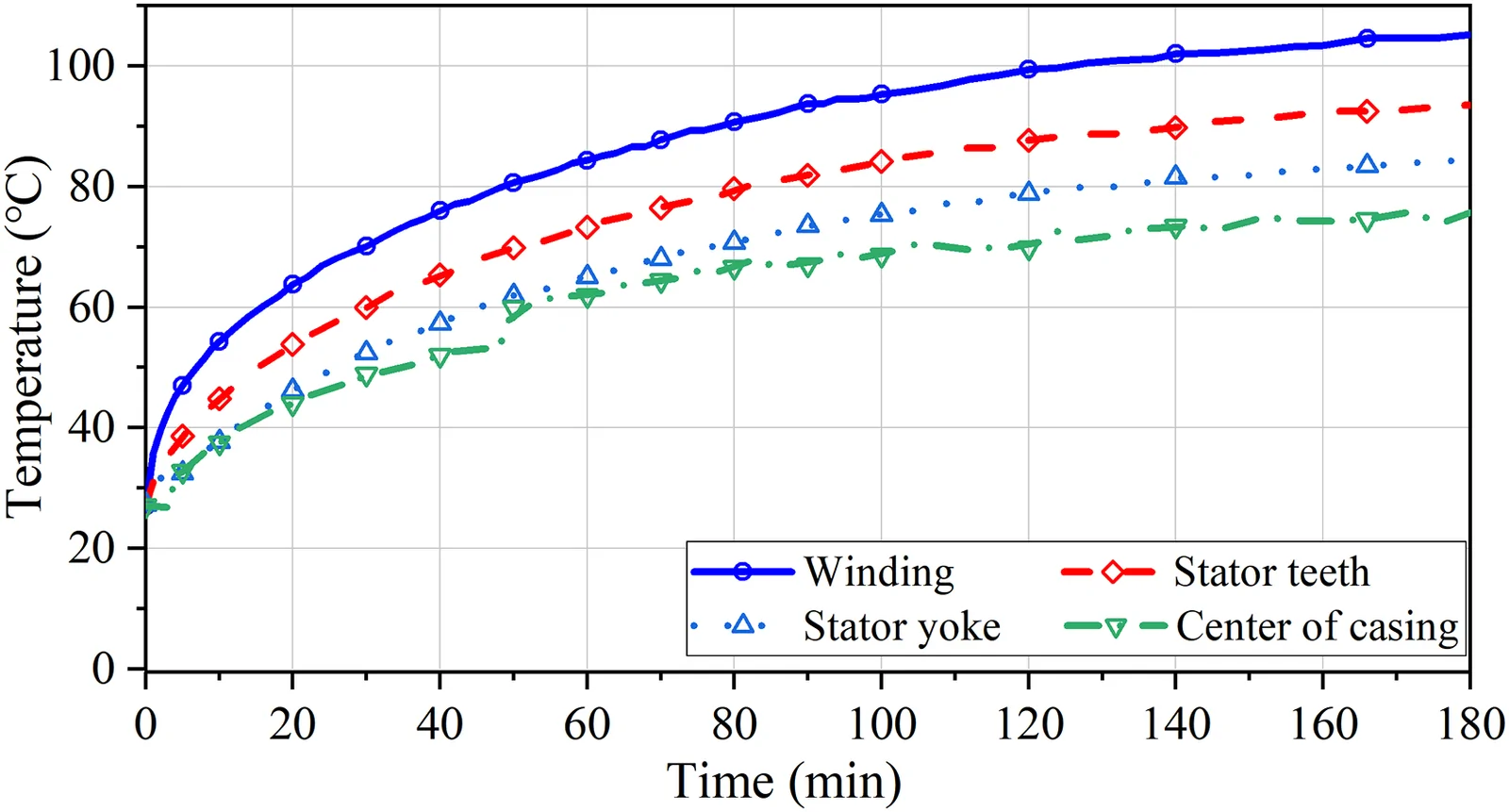
Temperature curve of the prototype under load condition at a current of 20A.

## 6. Discussion

The proposed hybrid laminated structure has a fan-shaped SMC matrix and embedded silicon steel sheets, which is fundamentally different from the previously cited hybrid and SMC assisted stator designs. Early work used only SMC stators [5–7], pure silicon steel stacked radially or axially [11,12], or empirical silicon steel/SMC ratios without geometric optimization [13]. In contrast, this work proposes a new integrated outer stator tooth topology that includes a quantified geometric formula for material ratios, placing high permeability silicon steel in the high-throughput saturation region while using SMC as the structural foundation. This uniquely design provides a thrust gain of 26% at the maximum displacement power stroke of the compressor, achieving previously unachievable target performance gains and manufacturing simplicity in hybrid structures.

In order to verify the suppression effect of stator segmentation on eddy current losses, the text analyzed the total damage of the hybrid laminated motor with different segmentation numbers using finite element method. Fig 19 shows the trend of total loss as a function of the number of stator tooth segments. As shown in the figure, when the number of stator tooth segments is 2, the total loss of the oscillating motor decreases to 16.04W. As the number of segments increases, the suppression effect on the total loss also decreases. This phenomenon is consistent with previous research findings [22]. When the number of stator tooth segments is 4, the total loss is 15.07W, which is less than the loss of a TPMLOA using only SMC material. Although the loss is greater than using traditional stacked TPMLOA, considering its improvement in thrust coefficient, this hybrid lamination method is still worth adopting.

**Fig 19:**
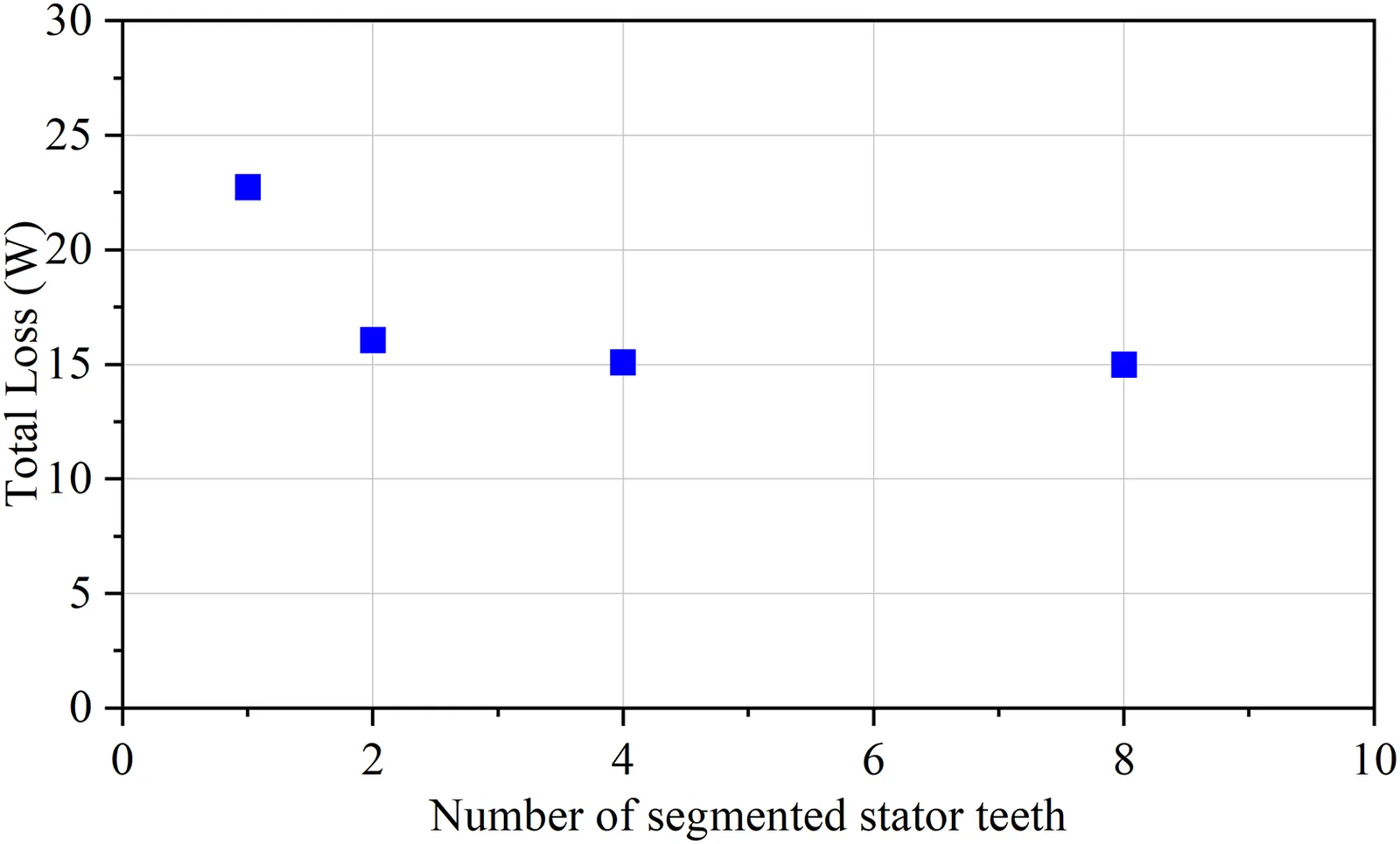
The trend of total loss changing with the number of stator tooth segments.

According to the characteristic structure and operating mode of TPMLOA, when the thrust and frequency are fixed, an increase in stroke leads to an increase in power. When the stroke is less than the rated stroke, the copper loss of the winding accounts for a relatively high proportion of the total loss. So as the stroke increases, the efficiency of the TPMLOA also increase. When the travel exceeds the rated time, not only does the eddy current loss increase rapidly, leading to a decrease in efficiency, but also the non-linear change area of the positioning force occurs, resulting in significant fluctuations in thrust and the risk of the mover hitting the bar. When the frequency and stroke remain constant, an increase in thrust leads to an increase in power. Efficiency increases with the increase of power. When the power exceeds the rated value, the saturation effect of the TPMLOA leads to a decrease in efficiency. At the same time, the increase in temperature also leads to an increase in copper loss, thereby reducing efficiency.

## 7. Conclusion

In this paper, a hybrid lamination method is proposed for the tubular permanent magnet linear oscillation actuator is proposed. The proportions of two materials in the outer stator are derived. Furthermore, the impact of the number of silicon steels in SMC material on the performance is discussed. Additionally, the effects of traditional radial lamination, SMC material and the hybrid lamination on the electromagnetic characteristics are compared using 3D FEA. Simulation and experimental results show that the hybrid lamination method proposed effectively reduces magnetic saturation in TPMLOA under load. In addition, this design has the best efficiency at 30–40 Hz and is suitable for small and medium-sized compressors, but has significant limitations at higher frequencies, power levels, and long strokes. Moreover, compared to a TPMLOA with only silicon steels, the TPMLOA using the hybrid lamination method increases electromagnetic thrust at the maximum mover displacement by 26%, with comparable thrust at the negative mover position. This targeted thrust improvement is especially beneficial for compressor applications, where high thrust at the maximum displacement power stroke is essential.
